# Comparative analysis of endophytic fungal communities in bamboo species *Phyllostachys edulis*, *Bambusa rigida*, and *Pleioblastus amarus*

**DOI:** 10.1038/s41598-023-48187-1

**Published:** 2023-11-27

**Authors:** Kuan Yan, Jian Zhang, Yu Cai, Guiling Cao, Lina Meng, Salma A. Soaud, Rania M. Y. Heakel, Muhammad Ihtisham, Xianming Zhao, Qin Wei, Tainfei Dai, Manzar Abbas, Ahmed H. El-Sappah

**Affiliations:** 1 Faculty of Agriculture, Forestry and Food Engineering, Yibin University, Yibin, China; 2 Sichuan Oil Cinnamon Engineering Technology Research Center, Yibin University, Yibin, China; 3 Genetics Department, Faculty of Agriculture, Zagazig University, Zagazig, 44511 Egypt; 4 Sichuan Green Food Development Center, Chengdu, 610041 China

**Keywords:** Genetics, Microbiology, Molecular biology, Plant sciences, Environmental sciences

## Abstract

Fungal endophytes in plant leaf mesophyll form mutually beneficial associations through carbon assimilation, synthesis of biologically active chemicals, and enhancement of aesthetic and nutritional value. Here, we compared community structure, diversity, and richness of endophytic fungi in the leaves of three bamboo species, including *Phyllostachys edulis* (MZ), *Bambusa rigida* (KZ), and *Pleioblastus amarus* (YT) via high-throughput Illumina sequencing. In total, 1070 operational taxonomic units (OTUs) were retrieved and classified into 7 phylum, 27 classes, 82 orders, 185 families, 310 genus, and 448 species. Dominant genera were *Cladosporium*, *Trichomerium*, *Hannaella*, *Ascomycota*, *Sporobolomyces*, *Camptophora* and *Strelitziana*. The highest fungal diversity was observed in *Pleioblastus amarus*, followed by *Bambusa rigida*, and *Phyllostachys edulis*. Comparatively, monopodial species *Ph. edulis* and sympodial *B. rigida*, mixed *P. amarus* revealed the highest richness of endophytic fungi. We retrieved a few biocontrol agents, *Sarocladium* and *Paraconiothyrium*, and unique *Sporobolomyces*, *Camptophora,* and *Strelitziana* genera. FUNGuild analysis revealed the surrounding environment (The annual average temperature is between 15 and 25 °C, and the relative humidity of the air is above 83% all year round) as a source of fungal accumulation in bamboo leaves and their pathogenic nature. Our results provide precise knowledge for better managing bamboo forests and pave the way for isolating secondary metabolites and potential bioactive compounds.

## Introduction

Endophytic microbiomes develop a kind of symbiotic association while residing inside different tissues and organs of plants^1^. Endophytic fungi constitute a dominant class of endophytes, which are ubiquitous for plant biotic and abiotic resistance^2^, enhancement of plant growth, improvement of nutritional value^3^, development of aesthetic value of food during pile-fermentation^4^, and biosynthesis of biologically active compounds such as phytohormones, secondary metabolites and phytotoxins in weeds^5,6^. Endophytic fungi promote plant growth by *mobilizing* nutrients, phosphate solubilization, and nitrogen fixation^5^. Diverse community structure and species richness of endophytic fungi in plants are predominantly defined by plant species, seed vector endophytes, life cycle, surrounding environment, and different plant tissues^7^. Among all tissues, diverse endophytes are predominantly accumulated in leaves due to their fragile nature, stomatal openings, airborne injuries, and easy transport^8^.

Diverse plant species generally host divergent endophytic fungal species^9^. Depth, analysis revealed that even different varieties of the same plant species also harbor diverse community structures and species richness of endophytic fungi^10^. For example, rhizomes collected from different varieties of moso bamboo located in different regions revealed significantly diverse endophytic microbiomes^11^. Similarly, tissue-specific analysis of *Eucalyptus globulus* and *Eucalyptus maidenii* revealed that sapwood, heartwood and bark harbor diverse colonies of endophytic fungi^12^. Intra-tissue-specific analysis of fungal colonies habituating leaves intramural environment of *Eucalyptus microcorys* revealed that fungal diversity was decreased but species richness was increased at different stages of growth^13^. In particular, a wide variety of endophytic fungal strains have been isolated from *Poaceae* plant family^6^.

*Bambusoideae* is a sub-family of *Poaceae*, with rich species diversity. There are more than 70 genera and more than 1200 species in the world^14^. Bamboo is a forest resource that integrates economic, ecological, and social benefits and plays an important role in modern forestry and regional economy. Bamboo stem is a rich source of natural, renewable, and biodegradable source of alternative of synthetic fiber i.e., cellulose macrofibres^15^. The strength of bamboo-derived cellulose macrofibres is 1.26 ± 0.21 GPa cm^−3^ g^−1^, stronger than steel, lightweight, and promising construction material for future elevated buildings to avoid the deteriorative effects of earthquakes^15^. Bamboo-derived thread has replaced petrochemical-based artificial fibers in the weaving industry^16^. In traditional medicines and cuisines, bamboo is being widely employed^17^. The endophytic microbes, particularly endophytic fungi play a pivotal role in the abundance of bamboo resources and derivatives^14,18^. In recent years, the diversity of endophytic fungi in different bamboo species has been investigated including *Phyllostachys praecox* and *Ph. edulis*^19^.

Investigation of endophytes is of great significance in the cultivation of bamboo forests, maintenance of bamboo forest ecosystems, and development of bamboo-based industry. Traditional culturing technique is not promising because numerous microbes are still unculturable. Next-generation sequencing techniques have made this possible to thoroughly investigate the microbiome of any species. Therefore, we employed a robust Illumina MiSeq™ PE300 platform to perform ITS sequencing to analyze the diversity and richness of endophytic fungal communities in bamboo leaves. Here, three densely populated verities of bamboo in Shunan Bamboo Sea, Yibin, China including *Phyllostachys edulis*, *Bambusa rigida* and *Pleioblastus amarus* were selected in this study. We first time reported a comprehensive and comparative analysis of community structure and diversity of endophytic fungal colons in the aforementioned three bamboo verities, which could be further employed in isolation of probiotics and instigation of bamboo growth.

## Materials and methods

### Collection of samples

In order to investigate endophytic fungi, leaves of three bamboo verities and three different sizes were collected including *Ph. edulis* (MZ), *B. rigida* (YT) and *P. amarus* (KZ) situated in Shunan Bamboo Sea (Yibin, Sichuan, 28°30′2″N, 105°04′7″E). To ensure maximum inclusion of endophytic fungal diversity, three biological replicates of each sample were selected with distantly separated. Healthy plants with vigorous growth were selected for collection of leaves and immediately stored at 4 °C to avoid mortality of endophytes. Upon arrival in the laboratory, samples were thoroughly washed with tap water to get rid of appendages and soil patches from the surface of leaves. Furthermore, 10 g of leaves were weighed with the help of electric balance and packed into sterile plastic bags, labelled, and stored at 4 °C for further DNA extraction and library construction for sequencing analysis. In order to avoid noise, surface sterilization of all samples was performed by soaking in 75% ethanol for 30 s followed by soaking in 2% NaClO solution for 2 min with gentle shaking on an agitator, and finally washed thrice with sterile ddH_2_O^20^. Confirmation of surface sterilization of leaves was performed by streaking of 20 ml of ddH_2_O of final washing on three petri plates filled with PDA medium. Plates were incubated at 28 °C in a biochemical incubator under dark conditions.

### DNA extraction

In order to extract genomic DNA, 5 g of each sample was chopped into very small pieces at 4 °C, and subsequently soaked in 25 ml of sterile ddH_2_O^21,22^. To remove coarse particles, each sample was filtered through a three-layered sterile gauze followed by centrifugation at 12,298×*g* for 10 min at 4 °C. Supernatant was discarded, and the filtrate was transferred into a new sterile tube for extraction of gDNA. Fungal genomic DNA was extracted with the help of E.Z.N.A™ Fungal DNA Miniprep Kit (OMEGA, USA) by following the standard protocol of the supplier. To confirm quality, 2 μL of gDNA was electrophoresed in 1% agarose gel, and visualized under UV light installed gel documentation system (iBright 1500, ThermoFisher, USA^22,23^. Furthermore, the concentration of high-quality gDNA was measured with the help of a spectrophotometer (Nanodrop 2000) at OD 260/280. The average concentration of gDNA of each sample was 2.1–42.5 ng/μL^24^.

### PCR amplification, library construction, and sequencing analysis

To amplify the ITS1 sequence of fungal ITS region with the help of PCR, the following universal primer pair ITS1F 5′CTTGGTCATTTAGAGGAAGTAA3′ and ITS2R 5′GCTGCGTTCTTCATCGATGC3′ was employed^3^. PCR reaction mixture was comprised of 1 PCR buffer, 1 mM dNTPs, 0.2 mM of each primer, 1.25 U of FastPfu DNA Polymerase (TransStart®, AP221-01, China), 2 ml DNA template, and a final volume of 25 mL was made by sterile ddH_2_O^25^. PCR conditions were: initial denaturation at 95 °C for 3 min, denaturation at 95 °C for 30 s, annealing at 55 °C for 45 s, amplification at 68 °C for 45 s, 32 cycles, final amplification at 68 °C for 20 min, and storage at 4 °C for infinity^26^. To confirm size of the amplicon, 2 μL of each PCR product was separated on 2% agarose gel and visualized under UV light^20^. Furthermore, a PCR product with the correct size was used to construct a gDNA library, and sequencing was performed at a high-fidelity Illumina MiSeq™ PE300 sequencing platform^7^.

Raw reads in fasta format were obtained from the sequencing of each sample. Reads of both MP or PE data were comprised of two files fq1 and fq2, reads of both sequencing ends and corresponding sequences. Furthermore, reads retrieved by the Illumina MiSeq® sequencing platform were comprised of paired-end sequence data. Incomplete raw reads were discarded, overlap sequences in PE pair-end reads were spliced into a full-length sequence, and duplicate reads were merged. To ensure the quality of sequencing data, filter analysis was performed and splicing events were measured with the help of Fast Length Adjustment of Short Reads (FLASH-1.2.11) software^27^. To avoid mixing of samples or any confusion, barcodes were generated on the base of primers and pasted on each sample before and after sequencing. We retrieved sufficient high-quality reads from each sample, their directions were corrected, and analyses were performed to disclose fungal diversity^28^.

### Phylogenetic clustering analysis of sequencing data

To identify OTUs in sequencing data to identify specific phyla of endophytic fungi, phylogenetic clustering analysis was performed by employing the Uparse v7.0.1090^1^ OTU clustering tool at an identity threshold level of 97%^29^. Chimeric sequences were identified and removed with the help of UCHIME v4.2^30^. Based on similarity index, sequences were clustered into bins known as Operational Taxonomic Units (OTUs)^31^. On the base of a reference library, the total number of combinations of different nucleotides were calculated at 100 bootstrap cutoff values. Prediction of taxonomy at the base of 80% *k*-mer similarity index was performed with the help of the SINTAX algorithm^32^. Endophytic fungal colonies were classified into kingdom, phylum, class, order, family, genus, and species^33^. Unique and common OTUs among all samples were counted and illustrated by constructing a Venn diagram^34^.

### Endophytic fungal diversity analysis

In order to estimate sequence coverage of both *α* and *β* diversity of endophytic fungi in bamboo leaves, Good’s coverage metric (C = 1 − n1/N) was employed, here “n1” represents the single sequence OTUs and “N” is a total number of sequences retrieved from one sample^35^. *α*-diversity of three biological repeats of each sample was calculated by employing Chao1, ACE, Shannon, and Simpson indices^36,37^. Euclidean distances, dissimilarity indexes, and diversity in OTUs at 97% identity threshold of all samples were calculated in each sample by principal coordinate analysis (PCoA) and non-metric multidimensional scaling (NMDS)^38^. FUNGuild database2 was explored to analyze functional groups of endophytic fungi present in bamboo leaves^39^. In-depth, comparative taxonomic analyses of endophytic fungal communities among all samples were performed at different levels of classification, and community structure diagrams and histograms were constructed in the R tool^40,41^.

### Statistical analysis

All readings were noted in mean values, and a one-way analysis of variance (ANOVA) was performed. Tukey’s HSD test was employed to further analyze variations among means of Shannon, Simpson, ACE, Chao, and diversity indices at a significance level of *p* < 0.05. All correlation and path coefficient analyses were performed with SPSS Statistics 20.0 software (SPSS Inc., Chicago, IL, United States) and Excel 2019.

### Statement

We certify that we obtained permission from the Yibin, Shunan Bamboo Sea Scenic Area to collect bamboo samples from *Phyllostachys edulis* (MZ), *Bambusa rigida* (KZ), and *Pleioblastus amarus* (YT) in the Shunan Bamboo Sea. The use of Moso bamboo in this study complied with all local, national, and international guidelines and regulations. We comply with the IUCN Policy Statement on Research Involving Species at Risk of Extinction and the Convention on the Trade in Endangered Species of Wild Fauna and Flora.

## Results

### Sequencing data analysis

Sequencing data analysis of endophytic fungal communities in all three samples of bamboo leaves has been presented in Table 1.

**Table 1:** Average internal transcribed spacer (ITS) reads of fungal endophytes in three species of Bamboo.

Sample	Reads	Total bases	Average length
MZ1	69,854	17,297,956	247.63
MZ2	98,122	24,508,382	249.77
MZ3	68,054	18,581,703	273.04
KZ1	50,532	12,455,406	246.49
KZ2	58,869	14,974,172	254.36
KZ3	62,181	14,763,573	237.43
YT1	67,548	18,155,099	268.77
YT2	52,446	14,156,954	269.93
YT3	46,708	10,994,863	235.40

*MZ *Ph. Edulis*, *KZ* *B. rigida*, *YT* *P. amarus.*

**Figure 1:**
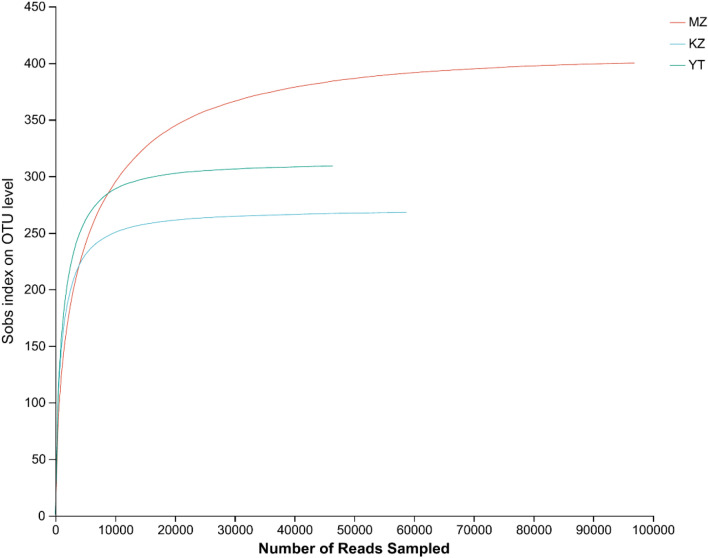
Rank abundance curves of all samples. The abscissa represents the rank number of operational taxonomic units (OTUs), and the ordinate represents the relative percentage of species at the classification level. The position of the abscissa of the extension end point of the sample curve represents the number of species in each sample. Smooth curves indicate higher species diversity, while steep decline indicates a high proportion of bacterial strains and low fungal diversity.

In sample MZ, complete reads free of noise were 69,854, 98,122, and 68,054, nucleotides were 17,297,956, 24,508,382, and 18,581,703 bp, and average lengths of sequences were 247.63, 249.77, and 273.04 bp. In sample KZ, the numbers of complete reads were 50,532, 58,869, and 62,181, total nucleotides were 12,455,406, 14,974,172, and 14,763,573 bp, and average lengths of sequences were 246.49, 254.36, and 237.43 bp. Similarly, the total number of reads in sample YT were 67,548, 52,446, and 46,708, while total nucleotides were 18,155,099, 14,156,954, and 10,994,863 bp, and average lengths of retrieved reads were 268.77, 269.93, and 235.40 bp. Rarefaction curves drawn from sequencing data revealed a flat pattern, which endorsed fidelity of sampling and number of sequencings to cover all taxa (Fig. 1). We observed a very high confidence level, which proved exact endophytic bacterial communities in all three samples of bamboo leaves (Fig. 1).

### Operational taxonomic unit (OTUs) cluster analysis

**Figure 2:**
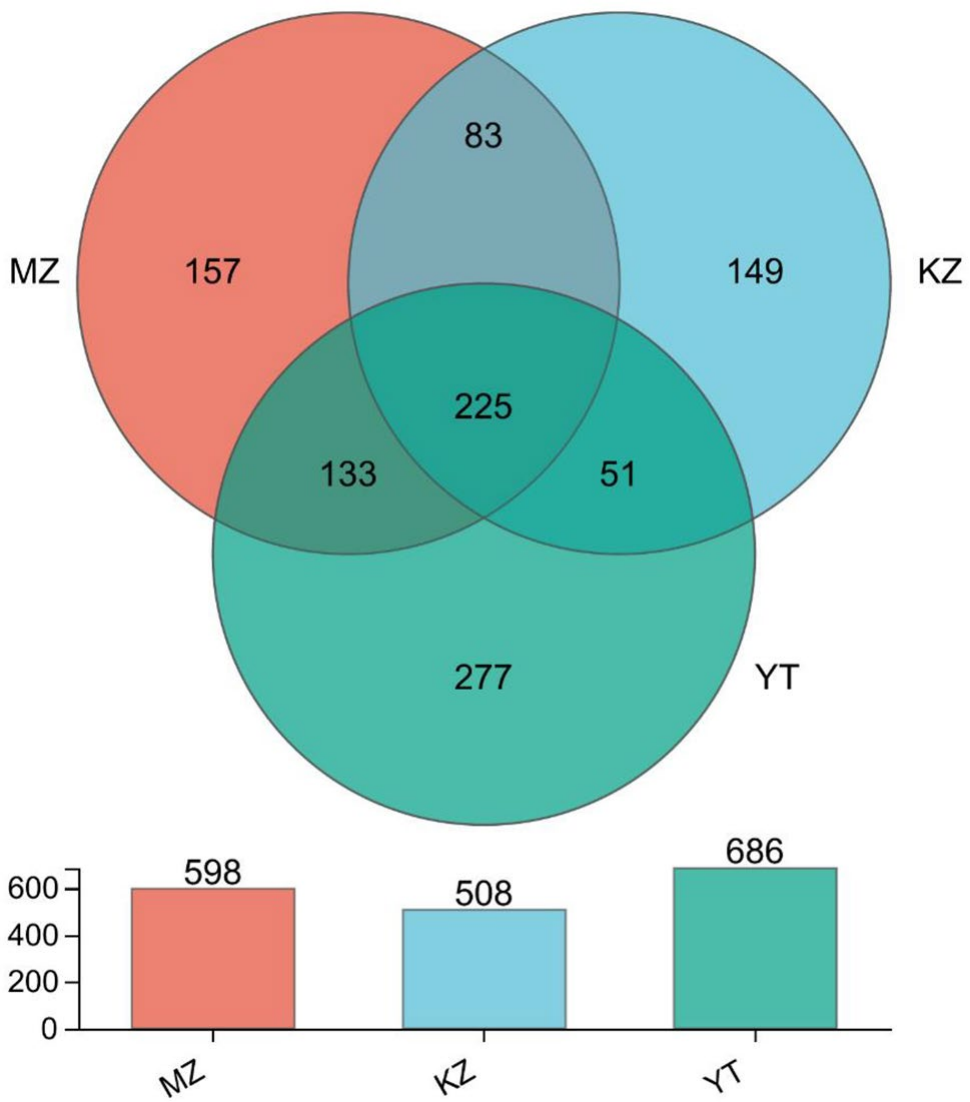
Venn diagram of OTUs. Different groups are represented by different colors, and numbers in overlapping portions represent the number of species common in all groups. *MZ *Ph. edulis*, *KZ*  *B. rigida*, *YT* *P. amarus.*

To show common and unique OTUs of endophytic fungi at a 97% identity threshold level in all three samples of bamboo leaves, a Venn diagram was constructed (Fig. 2). In total, 1070 OTUs were retrieved, which were further classified into 7 phylum, 27 classes, 82 orders, 185 families, 310 genera, and 448 species.

Total retrieved OTUs in individual samples MZ, KZ, and YT were 598, 508, and 686, respectively. Common OTUs between samples KZ and MZ were 83, between KZ and YT were 51, and between MZ and YT were 133. Unique OTUs in each sample MZ, KZ, and YT were 157 (26.3%), 149 (29.3%), and 277 (40.4%), respectively. Common OTUs among all three samples were 225 (12.6%). These results indicate a significant diversity of endophytic bacterial communities in all three samples of bamboo leaves (Fig. 2).

### Microbial abundance and diversity analysis

The sequencing depth of all three samples MZ, KZ, and YT were 0.9993, 0.9999, and 0.9997, respectively (Table 2). These results proved significant coverage of endophytic fungal communities in all three samples. We observed higher Shannon and lower Simpson indices in all three samples which also endorsed the highest diversity of fungal communities in all samples. Among all three samples, the highest diversity of endophytic fungal community was noted in the KZ sample, followed by YT, and lowest in MZ. Similarly, Chao and ACE indices revealed the highest richness of endophytic fungal communities in sample MZ, followed by YT, and lowest in sample KZ (Table 2). These results proved significant heterozygous abundance and diversity of endophytic fungi in all three bamboo species.

**Table 2:** Endophytic community richness and diversity indices of the three bamboo leaves.

Sample	Shannon	Simpson	ACE	Chao	Coverage
MZ	3.217 ± 0.315c	0.091 ± 0.024a	457.178 ± 17.457a	439.188 ± 34.319a	0.9993
KZ	4.073 ± 0.206a	0.045 ± 0.008c	285.113 ± 20.300c	262.500 ± 31.376c	0.9999
YT	3.679 ± 0.471b	0.085 ± 0.034b	319.105 ± 10.067b	321.310 ± 13.801b	0.9997

The last column contains the average values of the diversity index. Lowercase letters indicate significant differences between samples (p < 0.05). (*MZ *Ph. Edulis*, *KZ* *B. rigida*, *YT* P. amarus).

### Fungal community structure analysis

**Figure 3:**
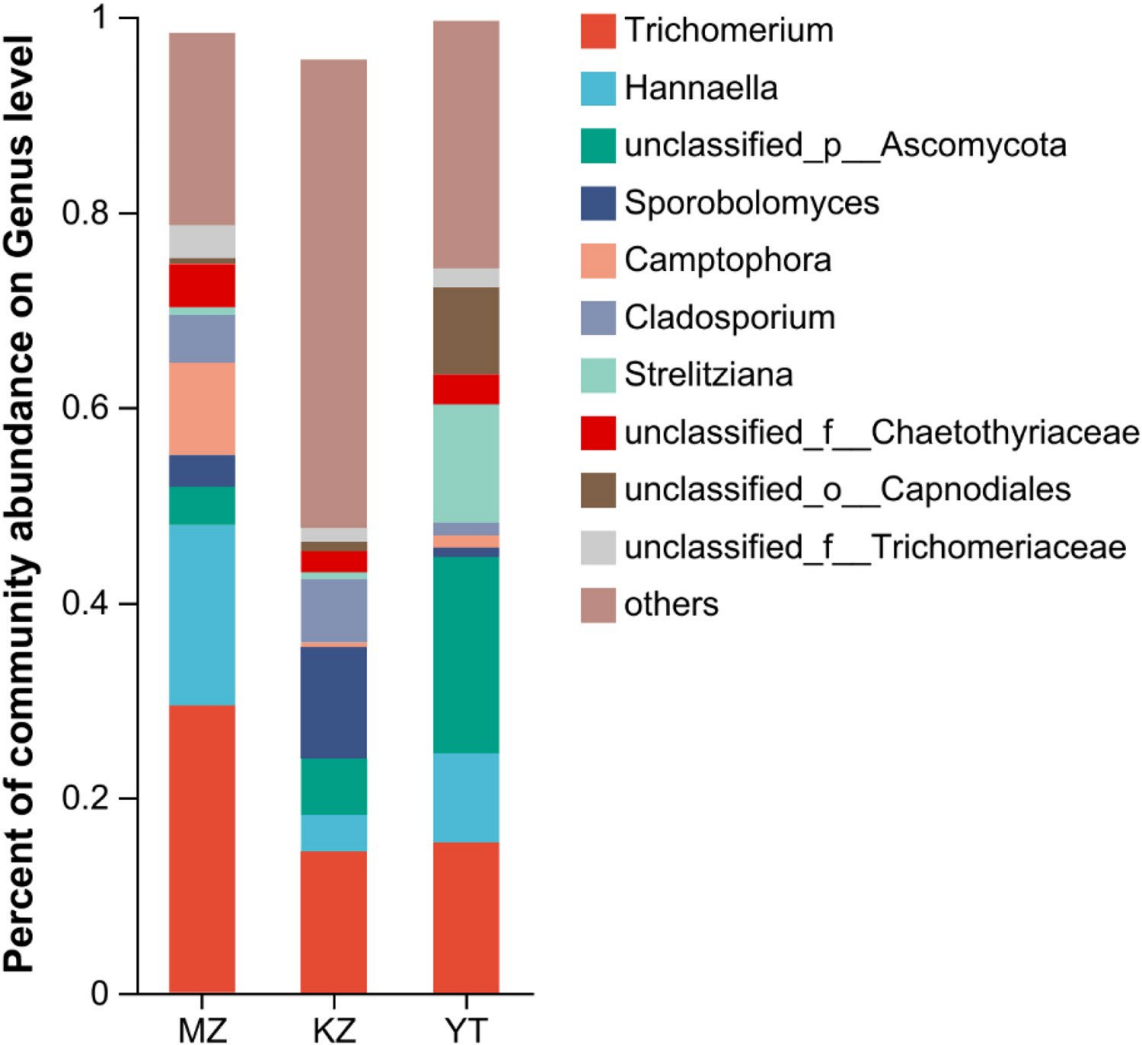
Fungal community structure bar plot analysis at the genus level. The ordinate represents the sample name, and the abscissa represents the percentage of different species, which is represented by columns with different colors, sizes, and proportions of a species. (*MZ *Ph. edulis*, *KZ* *B. rigida*, *YT* *P. amarus*).

In all three samples of bamboo leaves, highly abundant endophytic fungal OTUs were annotated and classified into 10 genera, and the rest of the OTUs were merged and classified as “others” (Fig. 3).

**Figure 4:**
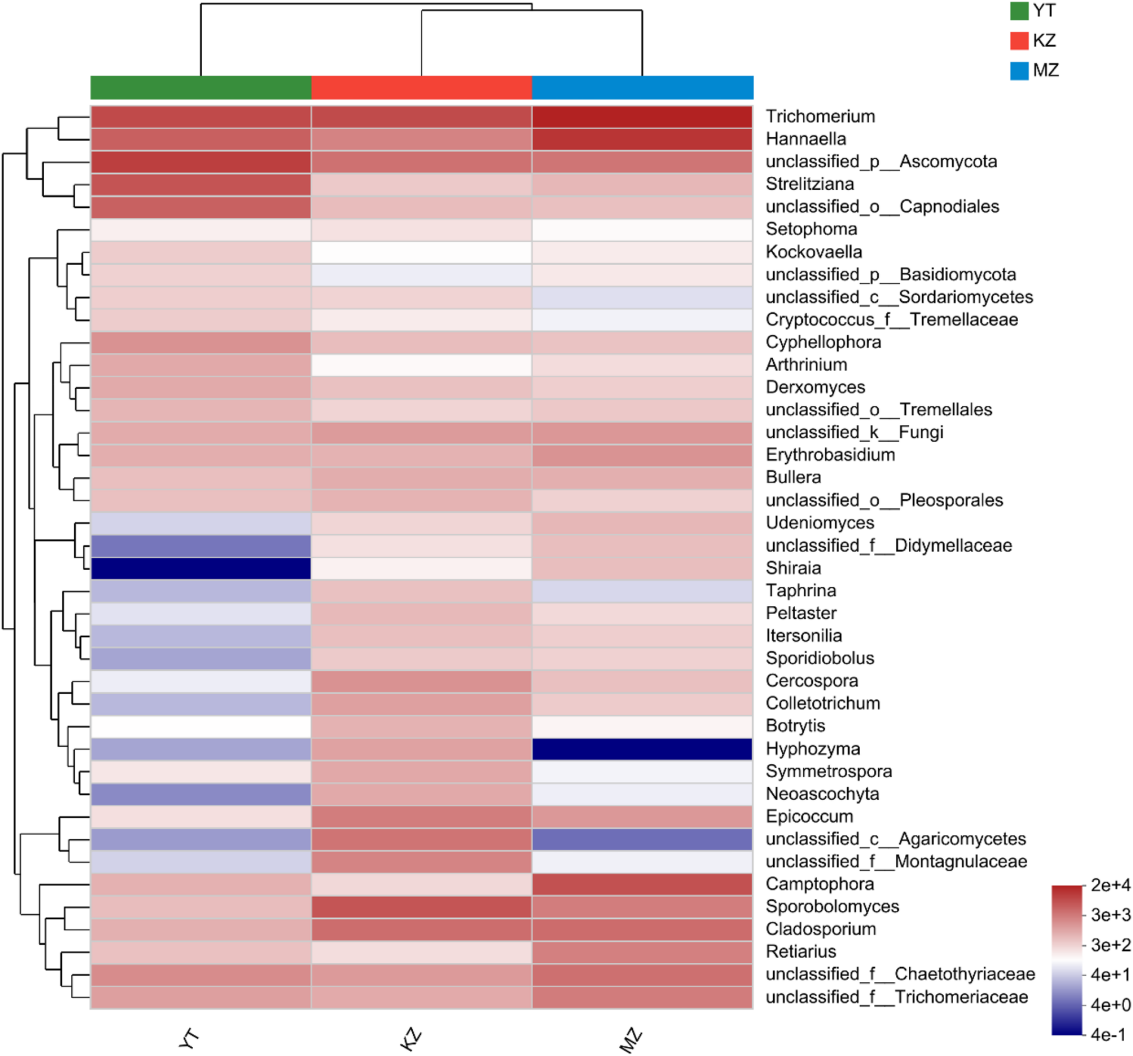
Community heatmap analysis of similarity in abundance between samples at the genus level. The bar on the right side represents the abundance value by color gradient. (*MZ *Ph. edulis*, *KZ* *B. rigida*, *YT* *P. amarus*).

Highly abundant endophytic genera in bamboo leaves are *Cladosporium*, *Trichomerium*, *Hannaella*, unclassified_p_*Ascomycota*, *Sporobolomyces*, *Camptophora,* and *Strelitziana* (Fig. 3). In precise manners, the percentage of highly abundant genera in sample MZ were *Trichomerium* (29.94%), *Hannaella* (18.78%), *Camptophora* (9.62%), *Cladosporium* (4.95%), and *unclassified_f__Chaetothyriaceae* (4.53%). Similarly, the percentage of dominant genera in sample KZ were *Trichomerium* (15.11%), *Sporobolomyces* (11.97%), *Cladosporium* (6.77%), *unclassified_p_Ascomycota* (6.02%), and *Hannaella* (3.93%). In sample YT, dominant genera were *unclassified_p_Ascomycota* (20.16%), *Trichomerium* (15.46%), *Strelitzia* (12.14%), *Hannaella* (9.20%), and *unclassified_o__Capnodiales* (8.90%). These findings endorsed the highest variation in abundance of endophytic fungi among three selected bamboo species. In all three samples of bamboo leaves, commonly highly expressed genera of endophytic fungi were *Trichomerium*, *Hannaella*, and *unclassified_p_Ascomycota* (Fig. 4). The highest richness of endophytic fungal genera was observed in sample YT, followed by MZ.

### *β*-Diversity analysis of endophytic fungal communities

**Figure 5:**
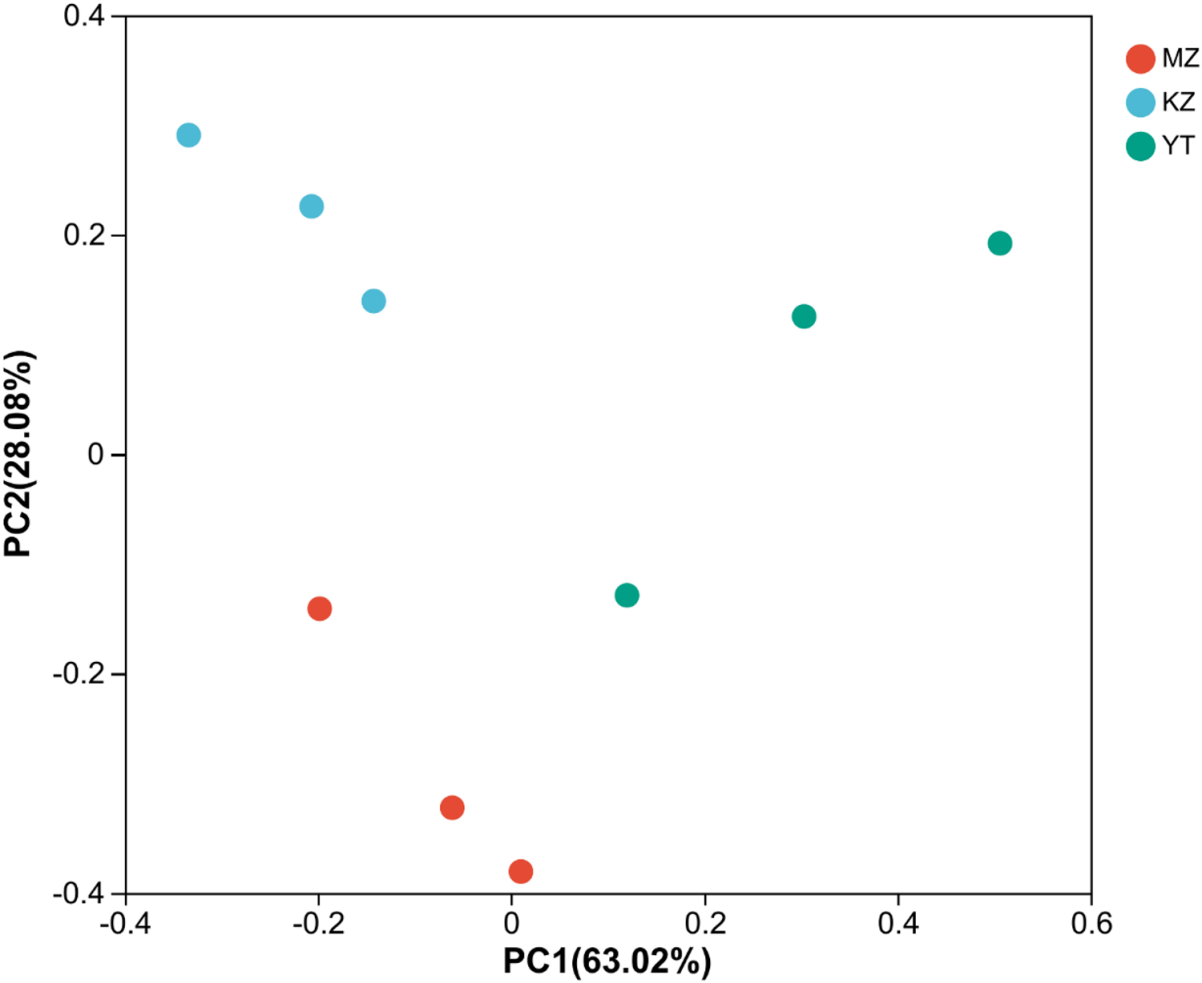
Multiple sample principal coordinate analysis (PCoA) of the OUT level. Both selected principal component axes are represented by the x-axis and y-axis, and the percentage represents the difference in sample composition by principal component; scales of the x-axis and y-axis represent relative distances. Samples are represented by different color points or shapes in different groups. The closeness of points or shapes represents the similarity level between fungal species composition. (*MZ *Ph. edulis*, *KZ* *B. rigida*, *YT* *P. amarus*).

**Figure 6:**
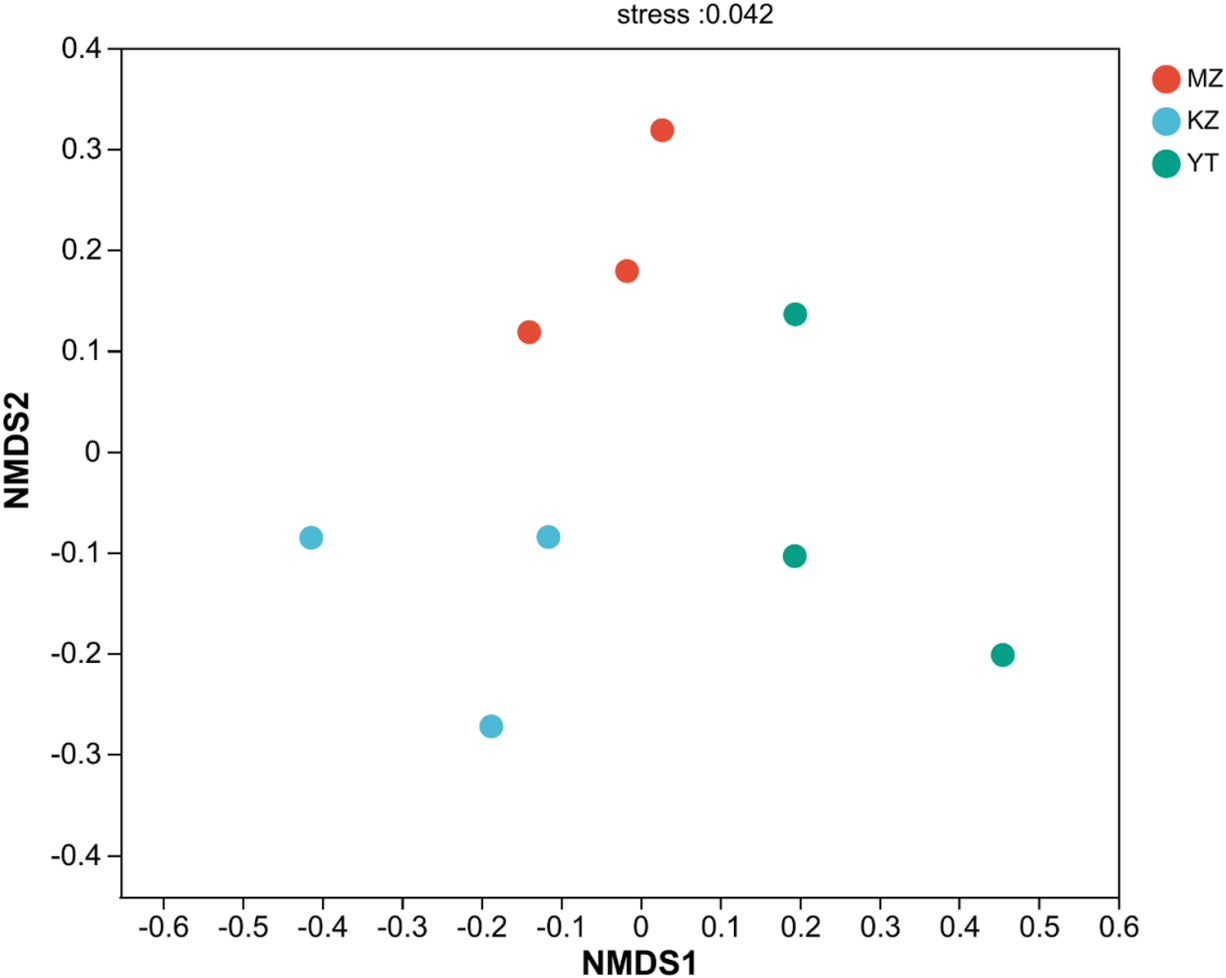
Samples are represented by different color points or shapes in different groups. The closeness of points or shapes represents the similarity level between fungal species composition. The abscissa represents the relative distance, which has no practical significance. Stress was used to examine the superiority of the results of NMDS analysis. It is generally considered that a two-dimensional dot plot representation of available NMDS when stress is < 0.2, representing its graph has some interpretive significance; When stress < 0.1, it can be considered a good ranking; When stress < 0.05, it indicates good representativeness. (*MZ *Ph. edulis*, *KZ* *B. rigida*, *YT* *P. amarus*).

To investigate intra-community diverse endophytic fungal species, *β*-diversity analysis was performed. Euclidean distances and variation index among all three samples were measured and illustrated via Principal Coordinate Analysis (PCoA) (Fig. 5). Highly abundant endophytic fungal genera in all samples were mapped to construct PCoA maps. The total of contributions of PC1 and PC2 in PCoA were 91.10% (Fig. 5). Similarly, non-metric multidimensional scaling (NMDS) analysis was performed to visualize pairwise dissimilarity multidimensional spaces in the form of low dimensional space for location, analysis, and classification. We observed that NMDS “stress” reached 0.042 (Fig. 6). Overall, the distances of all three bamboo samples were comparatively dispersed and further apart (Figs. 5 and 6).

These results proved the significant diversity among endophytic fungal communities in all three bamboo species.

### FUNGuild function prediction of endophytic fungi

**Figure 7:**
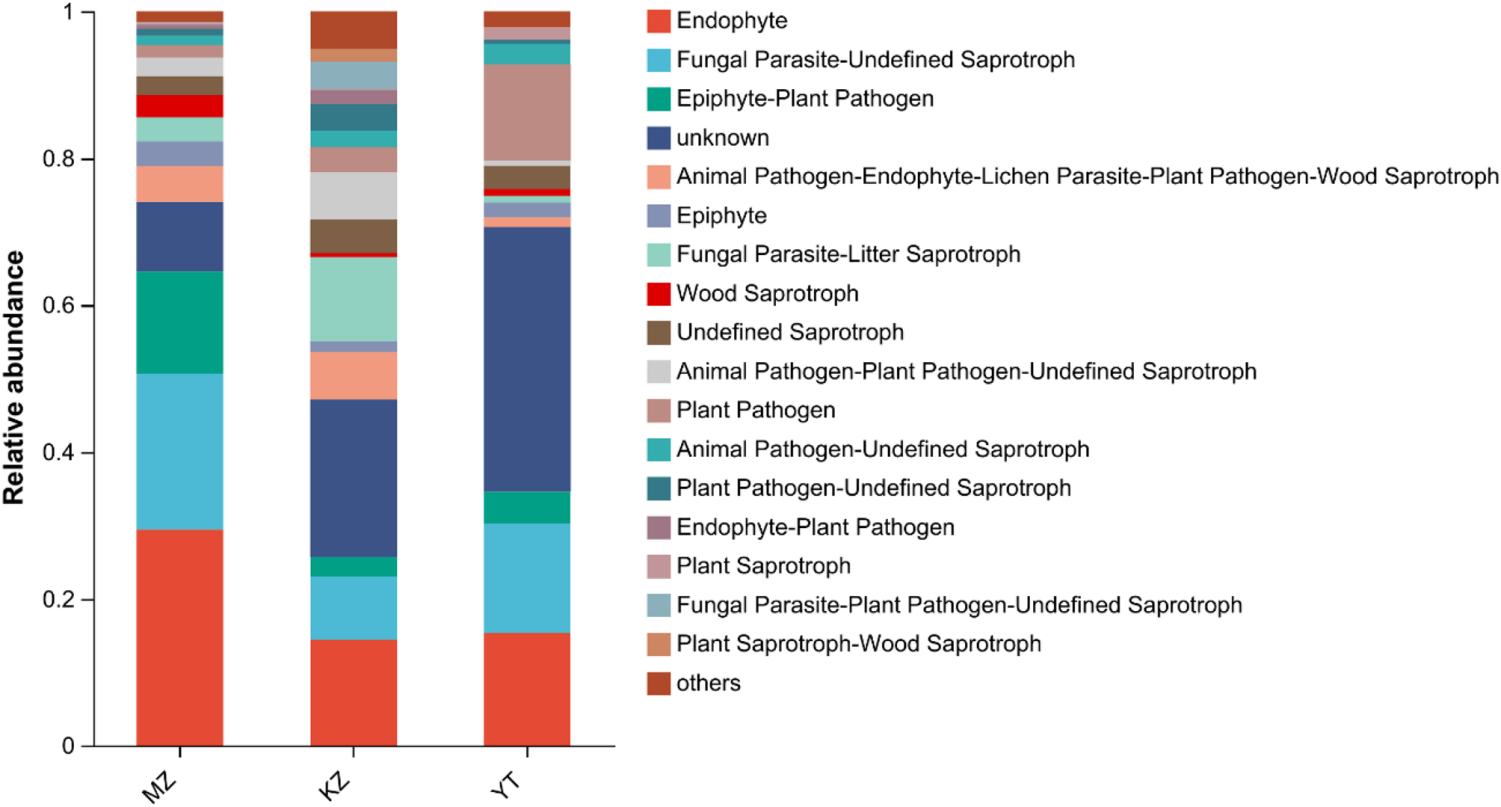
Functional Guild (FUNGuild) analysis of the fungal functional groups. Relative abundance of Guild in different groups or samples is depicted on the x-axis and groups or samples are presented on the y-axis. According to variation in the functional group, FUNGuild can calculate the abundance of each fungal species and its functional classification in each sample. (*MZ *Ph. edulis*, *KZ* *B. rigida*, *YT* *P. amarus*).

Fungi Functional Guild (FUNGuild) was employed to analyze endophytic fungal communities, their abundance among different samples, sources and involvement in specific pathways^42^. FUNGuild predicted functions of endophytic fungi in bamboo leaves as Endophyte, Fungal Parasite-Undefined Saprotroph, Epiphyte-Plant Pathogen, animal pathogen-endophyte-lichen parasite-plant pathogen-wood saprotroph, Epiphyte, Fungal Parasite-Litter Saprotroph, and Wood Saprotroph (Fig. 7). These results indicate that the dominant source of accumulation of endophytic fungi in bamboo leaves was the surrounding environment. The highest abundance of endophytic fungal species was accumulated in *P. amarus*, followed by *Ph. edulis* and *B. rigida*. The highest richness and diversity of endophytic fungi in bamboo leaves may a correlate with fast growth bamboo.

## Discussion

Endophytic fungi develop symbiotic relationships with bamboo plants to assist in nutrient accusation for fast growth, develop aroma and improve nutritional value during fermentation, and biosynthesis secondary metabolites^43^. Leaf mesophyll are the primary habitat of endophytes in plants where endophytes play a role in carbon fixation^44^. The genetic makeup, sprouting, growth time, growth potential and localization to specific regions are leading causes of endophytic fungal diversity, variable community structure, and their abundance in different bamboo species. For example, Yuan et al.^11^ found differences in the diversity of endophytic microorganisms among different varieties of *Populus*. Similar phenomena were also observed in the diversity of endophytic fungi in different varieties of *Rhodiola*^45^. We employed bamboo leaves to perform a comparative analysis of the community structure of endophytic fungi in three different bamboo species via robust high-throughput sequencing of ITS regions^46^. Diversity indexes of endophytic fungal communities among all three bamboo species were significantly different (Fig. 4), our findings are consistent with the diversity of endophytic fungi in the leaves of five different species of *Podocarpus*^47^. Highly heterologous community composition, diversity, and richness of endophytic fungi were observed in *Pleioblastus amarus* due to monopodial structure due to the sympodial structure of *Phyllostachys edulis*, and mixed *Bambusa rigida*^48,49^.

Study on different species of *Podocarpus* and *Tinospora* revealed that the geographical location and climatic conditions affect the endophytic fungal diversity in a given area^47^. Umali et al.^50^ investigated the endophytic fungi of bamboo species such as *Ph. edulis*, *Bambusa tuldoides*, *Drepanostachyum luodianense*, and *Phyllostachys heteroclada*, and found that most of these endophytic fungi belong to *Fusarium*, *Xylaria*, and *Arthrinium*. Morakotkarn et al.^51^ isolated 257 endophytic fungi from *Phyllostachys* and *Sasa* bamboo species, with *Arthrinium* being the main dominant fungal genus. Researchers have found that strains such as *Arthrinium*, *Pestalotiopsis*, *Shiraia*, and *Pleosporales* were isolated from six species of bamboo leaves^52^. In this study, *Ph. edulis*, *B. rigida,* and *P. amarus*, relatively abundant and common genera were *Trichomerium*, *Cladosporium*, *Hannaella*, and *unclassified_p_Ascomycota*, similar to *Aquilaira sinensis*^53^. *Sporobolomyces*, *Camptophora,* and *Strelitziana* are novel endophytic fungi first time identified in bamboo. A mutual relationship exists between plants and endophytes for their growth and the biosynthesis of secondary metabolites. Among them, *Trichoderma* is applied to biological control. Its advantage is that it can directly treat seeds or seedlings, avoiding the treatment of a large number of soil and adult plants^54^. Therefore, it has the advantages of sustainability, reducing pollution and cost. *Hannaella sinensis* is an effective biocontrol agent against apple blue mold decay^55^. *Cladosporium* was found in all species, while *Shiraia* was abundant in *P. amarus* and *Ph. edulis*, which are common biocontrol agents against bamboo blight, bamboo siderosis, and bamboo leaf rust^14^. *Cladosporium* was present in all three bamboo species which is a rich source of many biologically active compounds^56^. *Sarocladium* and *paraconiothyrium* are efficient biocontrol agents against root-knot nematode^57^. These potential biocontrol agents can be employed to control pathogens in bamboo forest forests.

The sooty blotch of bamboo is widely distributed among a variety of bamboo species across China^58^, which is causing serious loss in yield, ornamental, and economic value by affecting the rate of photosynthesis and respiration in bamboo^59^. The causative agent of sooty blotch in various bamboo species are variety of fungal pathogens that belong to the *Capnodiales* and *Meliolales* genera^60^, we observed significant contents of *Capnodiales* genus in *P. amarus* which need to be controlled for maximum yield with higher quality ornamental bamboo (Fig. 3). Following genera *Trichomerium*, *Sporobolomyces*, *Camptophora*, and *Strelitziana* were also abundantly found in all bamboo species (Figs. 3 and 4), which are involved in bamboo sooty blotch infection. These findings will be helpful in the timely management of pathogenic fungi to prevent bamboo plants from sooty blotch.

FUNGuild function prediction analysis revealed enrichment of fungi in bamboo leaves as Endophytes, Parasites, Epiphytes, Pathogens, and Saprotrophs (Fig. 7), which indicates that dominant fungi in all bamboo species are pathogenic. Under favorable conditions, the aforementioned pathogenic fungi could cause serious diseases or decay in bamboo plants such as sooty blotch^61^. Numerous plant diseases are caused by endophytic pathogens or seed vector pathogens, as observed in *Alpinia zerumbit* seeds^7^. Furthermore, highly abundant genera *Trichomerium*, *Sporobolomyces*, *Camptophora,* and *Strelitziana* in bamboo leaves can transform into pathogenic fungi and infest other plant species, so the comprehensive investigation is required to undermine possible devastating effects of fungi. FUNGuild analysis revealed a few incomplete enrichments of fungal pathogens, so further FUNGuild database improvement is also required.

## Conclusion

Bamboo is the world's fastest-growing forest resource, with numerous ecological, commercial, ornamental, and industrial advantages. Endophytic fungi live in many plant tissues, particularly leaf mesophyll cells, where they help to improve photosynthesis and the aesthetic and nutritional value of plants. Endophytic fungal communities in the leaves of the bamboo species *Phyllostachys edulis*, *Bambusa rigida*, and *Pleioblastus amarus* were studied using robust Illumina MiSeq™ high-throughput ITS sequences. Highly enriched endophytic fungal genera were *Cladosporium*, *Trichomerium*, *Hannaella*, *unclassified_p_Ascomycota*, *Sporobolomyces*, *Camptophora,* and *Strelitziana*. Here, we report discovery of three unique fungal genera in bamboo leaves for the first time. Biocontrol agents including *Sarocladium* and *Paraconiothyrium* were also identified in bamboo leaves. Proportion of diversity richness in three bamboo species were; *P. amarus* > *B. rigida* > *Ph. edulis*. FUNGuild analysis revealed the enrichment of dominant fungal species in pathogenesis.

### Supplementary Information

Supplementary Information.

## Data Availability

The datasets presented in this study can be found in online repositories. The names of the repository/repositories and accession number(s) can be found at: the European Nucleotide Archive (ENA), Accession number: PRJEB60242 (ERP145292).

## References

[1] Gond SK, Bergen MS, Torres MS, White JF (2015). Endophytic Bacillus spp. produce antifungal lipopeptides and induce host defence gene expression in maize. Microbiol. Res..

[2] Shi XJ (2021). Effects of endophyte infection on fungal disease resistance of *Achnatherum sibiricum* and non-symbiotic neighbours. Chin. J. Plant Ecol..

[3] Yan K (2021). Analysis of the fungal diversity and community structure in Sichuan dark tea during pile-fermentation. Front. Microbiol..

[4] Yan K (2021). Microbial Diversity in Sichuan Dark Tea During Pile-Fermentation.

[5] Donoso R (2017). Biochemical and genetic bases of indole-3-acetic acid (auxin phytohormone) degradation by the plant-growth-promoting rhizobacterium *Paraburkholderia phytofirmans* PsJN. Appl. Environ. Microbiol..

[6] Macías-Rubalcava ML, Garrido-Santos MY (2022). Phytotoxic compounds from endophytic fungi. Appl. Microbiol. Biotechnol..

[7] Yan K (2022). Determination of community structure and diversity of seed-vectored endophytic fungi in *Alpinia zerumbet*. Appl. Microbiol. Biotechnol..

[8] Cui J, Guo S, Xiao P (2017). Interaction between endophytes and host plant and the role of endophytes in genuineness analysis of medicinal plant. Acta Pharm. Sin..

[9] Liu J (2021). Microbial communities in rare earth mining soil after in-situ leaching mining. Sci. Total Environ..

[10] Massimo NC (2015). Fungal endophytes in aboveground tissues of desert plants: Infrequent in culture, but highly diverse and distinctive symbionts. Microb. Ecol..

[11] Yuan Z, Liu F, Zhang G (2014). Characteristics of endophytic bacteria isolated from mosobamboo rhizome and their 16S rDNA diversity. Genomics Appl. Biol..

[12] Sinneto S, Alonso R, Tiscornia S, Bettucci I (2005). Fungal community of Eucalyptus globulus and *Eucalyptus maidenii* stems in Uruguay. Sydowia.

[13] Lacerda LT, Gusmão LFP, Rodrigues A (2019). Fungal communities in different aged leaves of *Eucalyptus microcorys* F. Muell. Braz. J. Bot..

[14] Shen XY (2014). Diversity and antimicrobial activity of culturable endophytic fungi isolated from moso bamboo seeds. PLoS ONE.

[15] Li Z (2022). Sustainable high-strength macrofibres extracted from natural bamboo. Nat. Sustain..

[16] Nayak L, Mishra SP (2016). Prospect of bamboo as a renewable textile fiber, historical overview, labeling, controversies and regulation. Fashion Textiles..

[17] Nirmala C, Bisht MS, Laishram M (2014). Bioactive compounds in bamboo shoots: Health benefits and prospects for developing functional foods. Int. J. Food Sci. Technol..

[18] Zhou YJ, Li JH, Ross Friedman C, Wang HF (2017). Variation of soil bacterial communities in a chronosequence of rubber tree (*Hevea brasiliensis*) plantations. Front. Plant Sci..

[19] Zhang X (2019). Changes of root endophytic bacterial community along a chronosequence of intensively managed lei bamboo (*Phyllostachys praecox*) forests in subtropical China. Microorganisms..

[20] El-Sappah AH (2021). Comprehensive genome wide identification and expression analysis of MTP gene family in tomato (*Solanum lycopersicum*) under multiple heavy metal stress. Saudi J. Biol. Sci..

[21] Yan K (2022). Determination of community structure and diversity of seed-vectored endophytic fungi in Alpinia zerumbet. Front. Microbiol..

[22] El-Sappah A (2022). Identification of novelroot-knot nematode (*Meloidogyne incognita*) resistant tomato genotypes. J. Anim. Plant Sci..

[23] El-Sappah AH (2023). Genome-wide identification and expression analysis of metal tolerance protein (MTP) gene family in soybean (Glycine max) under heavy metal stress. Mol. Biol. Rep..

[24] El-Sappah AH, Shawky A, Sayed-Ahmad MS, Youssef M (2017). Estimation of heat shock protein 70 (hsp 70) gene expression in nile tilapia (*Oreochromis niloticus*) using quantitative Real-Time PCR. Zagazig J. Agric. Res..

[25] Wang J (2018). Selection and validation of reference genes for quantitative gene expression analyses in black locust (*Robinia pseudoacacia* L.) using real-time quantitative PCR. PLoS ONE..

[26] Abbas M (2020). Involvement of *CesA4*, *CesA7-A/B* and *CesA8-A/B* in secondary wall formation in *Populus trichocarpa* wood. Tree Physiol..

[27] Sanders JG (2019). Optimizing sequencing protocols for leaderboard metagenomics by combining long and short reads. Genome Biol..

[28] Mukherjee PK (2014). Oral mycobiome analysis of HIV-infected patients: Identification of *Pichia* as an antagonist of opportunistic fungi. PLoS Pathog..

[29] Ye J (2017). Chemolithotrophic processes in the bacterial communities on the surface of mineral-enriched biochars. ISME J..

[30] Edgar RC, Haas BJ, Clemente JC, Quince C, Knight R (2011). UCHIME improves sensitivity and speed of chimera detection. Bioinformatics..

[31] Nguyen N-P, Warnow T, Pop M, White B (2016). A perspective on 16S rRNA operational taxonomic unit clustering using sequence similarity. NPJ Biofilms Microb..

[32] Edgar RC (2016). SINTAX: A simple non-Bayesian taxonomy classifier for 16S and ITS sequences. Biorxiv..

[33] Chen B (2016). Biodiversity and activity of the gut microbiota across the life history of the insect herbivore *Spodoptera littoralis*. Sci. Rep..

[34] Fouts DE, Brinkac L, Beck E, Inman J, Sutton G (2012). PanOCT: Automated clustering of orthologs using conserved gene neighborhood for pan-genomic analysis of bacterial strains and closely related species. Nucleic Acids Res..

[35] Phan-Minh, T., Grigore, E. C., Boulton, F. A., Beijbom, O. & Wolff, E. M. in *Proceedings of the IEEE/CVF Conference on Computer Vision and Pattern Recognition.* 14074–14083. 10.48550/arXiv.1911.10298 (2020).

[36] Gihring TM, Green SJ, Schadt CW (2012). Massively parallel rRNA gene sequencing exacerbates the potential for biased community diversity comparisons due to variable library sizes. Environ. Microbiol..

[37] Rogers MB (2016). Disruption of the microbiota across multiple body sites in critically ill children. Microbiome..

[38] Calderón K (2017). Effectiveness of ecological rescue for altered soil microbial communities and functions. ISME J..

[39] Song H (2019). Tropical forest conversion to rubber plantation in southwest China results in lower fungal beta diversity and reduced network complexity. FEMS Microbiol. Ecol..

[40] Lu Y (2016). Mucosal adherent bacterial dysbiosis in patients with colorectal adenomas. Sci. Rep..

[41] Zhou YK, Shen XY, Hou CL (2017). Diversity and antimicrobial activity of culturable fungi from fishscale bamboo (*Phyllostachys heteroclada*) in China. World J. Microbiol. Biotechnol..

[42] Schmidt R, Mitchell J, Scow K (2019). Cover cropping and no-till increase diversity and symbiotroph: Saprotroph ratios of soil fungal communities. Soil Biol. Biochem..

[43] Amara AA, El-Baky NA (2023). Fungi as a source of edible proteins and animal feed. J. Fungi..

[44] Suryanarayanan T, Ayesha M, Shaanker RU (2022). Leaf photosynthesis: Do endophytes have a say?. Trends Plant Sci..

[45] Cui J-L, Guo T-T, Ren Z-X, Zhang N-S, Wang M-L (2015). Diversity and antioxidant activity of culturable endophytic fungi from alpine plants of *Rhodiola crenulata*, *R. angusta*, and *R. sachalinensis*. PLoS ONE..

[46] Wani GA, Khan MA, Dar MA, Shah MA, Reshi ZA (2021). Next generation high throughput sequencing to assess microbial communities: An application based on water quality. Bull. Environ. Contamin. Toxicol..

[47] Joshee S, Paulus BC, Park D, Johnston PR (2009). Diversity and distribution of fungal foliar endophytes in New Zealand Podocarpaceae. Mycol. Res..

[48] Li Y (2022). Comparison of bacterial and fungal community structure and potential function analysis of yak feces before and after weaning. BioMed Res. Int..

[49] Gao J (2019). Comparison on structure and diversity of endophytic bacteria between three species of bamboo. J. Bamboo Res..

[50] Umali TE, Quimio TH, Hyde KD (1999). Endophytic fungi in leaves of *Bambusa tuldoides*. Fungal Sci..

[51] Morakotkarn D, Kawasaki H, Seki T (2007). Molecular diversityof bamboo-associated fungi isolated from Japan. FEMS Microbiol. Lett..

[52] Xu X, Bai Y, Liu H, Yang C, Liu Y (2014). Microform analysis on six kinds of bamboo leaves in Sichuan Province. J. Huazhong Agric. Univ..

[53] Du T-Y (2022). Endophytic fungi associated with *Aquilaria sinensis* (Agarwood) from China show antagonism against bacterial and fungal pathogens. J. Fungi..

[54] Wani ZA, Ashraf N, Mohiuddin T, Riyaz-Ul-Hassan S (2015). Plant-endophyte symbiosis, an ecological perspective. Appl. Microbiol. Biotechnol..

[55] Lin R (2022). Study on the biocontrol effect and physiological mechanism of *Hannaella sinensis* on the blue mold decay of apples. Int. J. Food Microbiol..

[56] Salvatore MM, Andolfi A, Nicoletti R (2021). The genus Cladosporium: A rich source of diverse and bioactive natural compounds. Molecules..

[57] Bhat AA, Shakeel A, Waqar S, Handoo ZA, Khan AA (2023). Microbes vs nematodes: Insights into biocontrol through antagonistic organisms to control root-knot nematodes. Plants..

[58] Huang L, He J, Tian C-M, Li D-W (2023). Bambusicolous fungi, diseases, and insect pests of bamboo. For. Microbiol..

[59] Belding R, Sutton T, Blankenship S (1994). Relationship between the surface wax of apple fruit and sooty blotch disease. HortScience..

[60] Liu WY (2020). Influence of sooty blotch on photosynthetic and eco-physiological characteristics of *Phyllostachys nigra* var. henonis. For. Res..

[61] Song Z, Kennedy PG, Liew FJ, Schilling JS (2017). Fungal endophytes as priority colonizers initiating wood decomposition. Funct. Ecol..

